# Targeting Mitochondrial Dynamics during Lower-Limb Ischemia Reperfusion in Young and Old Mice: Effect of Mitochondrial Fission Inhibitor-1 (mDivi-1)

**DOI:** 10.3390/ijms25074025

**Published:** 2024-04-04

**Authors:** Stéphanie Paradis, Anne-Laure Charles, Margherita Giannini, Alain Meyer, Anne Lejay, Samy Talha, Gilles Laverny, Anne Charloux, Bernard Geny

**Affiliations:** 1 Biomedicine Research Center of Strasbourg (CRBS), UR 3072, “Mitochondria, Oxidative Stress and Muscle Plasticity”, Faculty of Medicine, University of Strasbourg, 67081 Strasbourg, France; stephanie.paradis@univ-grenoble-alpes.fr (S.P.); anne.laure.charles@unistra.fr (A.-L.C.); margherita.giannini@chru-strasbourg.fr (M.G.); alain.meyer1@chru-strasbourg.fr (A.M.); anne.lejay@chru-strasbourg.fr (A.L.); samy.talha@chru-strasbourg.fr (S.T.); anne.charloux@chru-strasbourg.fr (A.C.); 2 Department of Physiology and Functional Explorations, University Hospital of Strasbourg, 67000 Strasbourg, France; 3 Vascular Surgery Department, University Hospital of Strasbourg, 67000 Strasbourg, France; 4 Institut de Génétique et de Biologie Moléculaire et Cellulaire (IGBMC), 67400 Illkirch, France; laverny@igbmc.fr

**Keywords:** peripheral arterial disease, ischemia reperfusion, muscle, aging, mitochondria, reactive oxygen species, mitochondrial dynamics, lactate

## Abstract

Peripheral arterial disease (PAD) strikes more than 200 million people worldwide and has a severe prognosis by potentially leading to limb amputation and/or death, particularly in older patients. Skeletal muscle mitochondrial dysfunctions and oxidative stress play major roles in this disease in relation with ischemia-reperfusion (IR) cycles. Mitochondrial dynamics through impairment of fission–fusion balance may contribute to skeletal muscle pathophysiology, but no data were reported in the setting of lower-limb IR despite the need for new therapeutic options. We, therefore, investigated the potential protective effect of mitochondrial division inhibitor-1 (mDivi-1; 50 mg/kg) in young (23 weeks) and old (83 weeks) mice submitted to two-hour ischemia followed by two-hour reperfusion on systemic lactate, muscle mitochondrial respiration and calcium retention capacity, and on transcripts specific for oxidative stress and mitochondrial dynamics. At the systemic levels, an IR-related increase in circulating lactate was still major despite mDivi-1 use (+305.9% *p* < 0.0001, and +269.4% *p* < 0.0001 in young and old mice, respectively). Further, IR-induced skeletal muscle mitochondrial dysfunctions (more severely impaired mitochondrial respiration in old mice (OXPHOS CI state, –68.2% *p* < 0.0001 and −84.9% *p* < 0.0001 in 23- and 83-week mice) and reduced calcium retention capacity (–46.1% *p* < 0.001 and −48.2% *p* = 0.09, respectively) were not corrected by mDivi-1 preconditioning, whatever the age. Further, mDivi-1 treatment did not oppose superoxide anion production (+71.4% *p* < 0.0001 and +37.5% *p* < 0.05, respectively). At the transcript level, markers of antioxidant enzymes (SOD 1, SOD 2, catalase, and GPx) and fission markers (Drp1, Fis) remained unchanged or tended to be decreased in the ischemic leg. Fusion markers such as mitofusin 1 or 2 decreased significantly after IR in both groups. In conclusion, aging enhanced the deleterious effects or IR on muscle mitochondrial respiration, and in this setting of lower-limb IR, mDivi-1 failed to protect the skeletal muscle both in young and old mice.

## 1. Introduction

Peripheral arterial disease (PAD), which usually develops on the basis of atherosclerosis and the narrowing of the arteries in the lower extremities, causes repeated ischemia-reperfusion (IR) cycles affecting skeletal muscles of the limb, resulting in lifestyle-limiting disability, including rest pain, exercise intolerance, and in the most severe cases, limb amputation. PAD is a major public health concern, with more than 200 million people diagnosed in the world, and is often associated with other cardiovascular diseases, including myocardial infarction, stroke, and cognitive dysfunctions.

Revascularization is not the first-choice option in asymptomatic PAD or claudication, since exercise therapy is preferred. However, in some cases such as critical limb ischemia, revascularization is required for limb salvage. 

Further, revascularization is complemented with exercise and pharmacotherapy and improves the functional status and clinical outcome in PAD patients [1,2,3,4,5,6,7,8,9].

However, improved therapeutic options based on PAD pathophysiology are still needed. 

Growing evidence suggests that skeletal muscle mitochondrial dysfunction and oxidative stress play major roles in the disease manifestation and decline of patients suffering from PAD, because alteration of oxidative phosphorylation capacity hampers energy generation and increases oxidative stress, promoting cell damage and death. Particularly, although indispensable, the reperfusion results in mitochondrial Ca^2+^ overload and the early opening of the mitochondrial permeability transition pore (mPTP) leads to severe muscle damage [10,11,12,13,14,15,16].

Interestingly, further underlining the importance of mitochondria, mitochondrial content in skeletal muscle demonstrated to be a good predictor of the mortality rate during PAD in humans and mitochondrial dysfunctions participate in PAD-associated sarcopenia [17,18]. Thus, improving our knowledge of the mitochondrial implication during IR appears mandatory.

Experimental models demonstrated various effects concerning limb protection when using ischemic or pharmacological pre- and post-conditioning [6,19], but mitochondrial dynamic modulation might be promising since it plays a critical role in controlling mitochondrial morphology and energetic metabolism [20,21,22,23]. Mitochondrial fission modulation with the mitochondrial division inhibitor-1 (mDivi-1), known as an inhibitor of dynamin-related protein 1 (Drp1) function, demonstrated protective effects in the setting of IR in several organs, including kidneys, brain, and heart [24,25,26,27,28,29,30]. On the other hand, Ong et al. recently demonstrated that using mDivi-1 failed to reduce myocardial infarction size or to preserve left ventricular function in pigs submitted to coronary artery ligation [31].

Although the impairment of mitochondrial quality control related to fission–fusion abnormalities may contribute to muscle alterations in the setting of cardiac or hindlimb IR [32,33], to the best of our knowledge, there is no study aiming to modulate the mitochondrial dynamics during lower-limb IR in young and old mice. 

The aim of this study was, therefore, to investigate such new therapeutic options, and we determined whether modulation of mitochondrial fission with the administration of mDivi-1 might protect skeletal muscle, improving, thus, mitochondrial respiration and calcium retention capacity (CRC), and reducing oxidative stress through decreased reactive oxygen species (ROS) production and/or stimulated antioxidant system. Since, IR is often more deleterious in young versus aged muscles, and since old mitochondria are more sensitive to several toxins such as alcohol and cannabis than younger ones [34,35], we analyzed the mitochondrial muscular responses to IR both in young and old animals using mDivi-1 as pharmacological preconditioning.

## 2. Results

### 2.1. Effects of IR on Systemic Lactate in mDivi-1-Treated Young and Old Mice

As expected, ischemia increased systemic lactate and reperfusion allowed a return toward baseline values.

**Figure 1:**
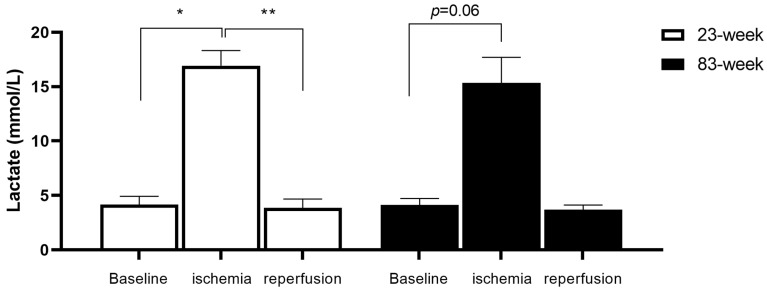
Kinetic of systemic lactate during lower-limb ischemia reperfusion. *: *p* < 0.05, **: *p* < 0.01.

In the young (23 weeks) group, lactate increased at the end of 2 h of ischemia (4.17 ± 0.75, 16.93 ± 1.40 mmol/L, *p* < 0.05) and returned toward baseline value at the end of reperfusion (3.86 ± 0.82 mmol/L, *p* < 0.01, Figure 1). 

A similar evolution was observed in the old (83 weeks) group and lactate increased at the end of 2 h of ischemia (4.15 ± 0.57 vs. 15.33 ± 2.35 mmol/L, *p* = 0.06) and decreased at the end of the reperfusion, (3.70 ± 0.42 mmol/L).

Results are presented as mean ± SEM. Lactate is measured just before starting ischemia (baseline) and, thereafter, at the end of 2 h of ischemia and at the end of the 2 h of reperfusion, respectively. *: *p* < 0.05, **: *p* < 0.01 compared to ischemia group.

### 2.2. Effects of IR on Mitochondrial Respiration and Calcium Retention Capacity in mDivi-1-Treated Young and Old Mice

#### 2.2.1. Mitochondrial Respiration

**Figure 2:**
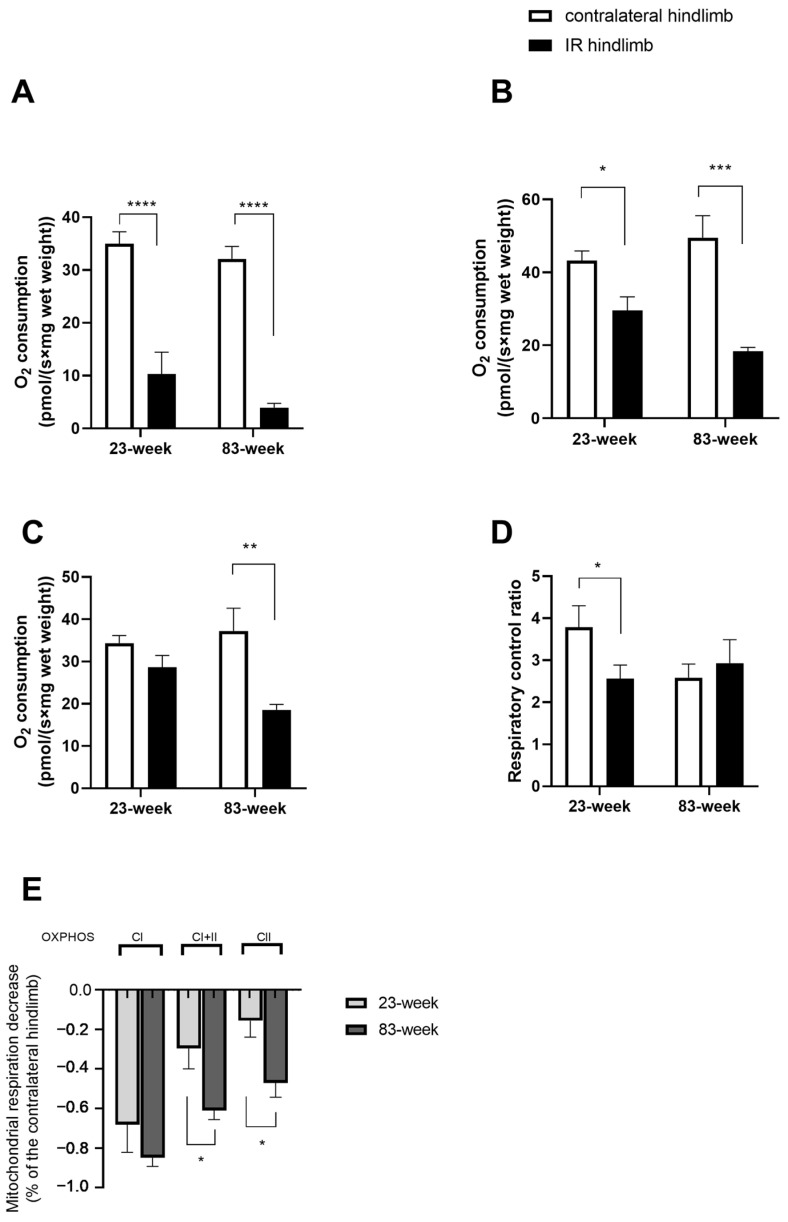
Effects of IR on mitochondrial respiration in mDivi-1-treated young and old mice. (**A**) The OXPHOS CI state with the addition of adenosine diphosphate (ADP). (**B**) OXPHOS CI + II with the addition of succinate. (**C**) OXPHOS CII with the addition of rotenone. (**D**) Respiratory control ratio (RCR), V_ADP_/V_0_ ratio. (**E**) Comparison between the 2 age groups of the extent of alteration, expressed in % of the contralateral hindlimb. Results are presented as mean ± SEM. *: *p* < 0.05, **: *p* < 0.01, ***: *p* < 0.001, ****: *p* < 0.0001. IR: ischemia reperfusion.

In the young population (23 weeks), ischemia reperfusion (IR) altered the oxidative phosphorylation (OXPHOS) complex I (CI) rate compared to the contralateral hindlimb (10.28 ± 4.16 vs. 34.98 ± 2.25 pmol/(s×mg wet weight), *p* < 0.0001, Figure 2A). This impairment is also observed in the OXPHOS CI + II rate with 29.58 ± 3.70 and 43.22 ± 2.66 pmol/(s×mg wet weight), *p* < 0.0001, Figure 2B.

When the complex I was inhibited by rotenone, IR tended to decrease the mitochondrial respiration (Figure 2C).

Finally, the respiratory control capacity (i.e., V_ADP_/V_0_ ratio), representing the degree of coupling between oxidation and phosphorylation, is significantly decreased with IR (2.56 ± 0.31 and 3.78 ± 0.52, *p* < 0.05 Figure 2D). 

In the older population (83 weeks), IR decreased significantly the OXPHOS CI rate compared to the contralateral hindlimb: 3.97 ± 0.79 vs. 32.11 ± 2.37 pmol/(s×mg wet weight), *p* < 0.0001 (Figure 2A).

For OXPHOS CI + II, the mitochondrial respiration is also impaired significantly: 18.30 ± 1.10 vs. 49.51 ± 6.02 pmol/(s×mg wet weight), *p* < 0.001 (Figure 2B).

Similarly, OXPHOS CII also decreased: 18.57 ± 1.26 vs. 37.21 ± 5.41 pmol/(s×mg wet weight), *p* < 0.01 (Figure 2C).

Finally, the RCR is not altered in the older population (Figure 2D).

When comparing these impairments in both populations, the alteration is significantly more severe in the older group of mice, particularly considering complex II (Figure 2E). For OXPHOS CI + II, the decrease is −29.63 ± 0.10 and 61.06 ± 4.56% for young and old mice group, respectively, with *p* < 0.05.

And for OXPHOS CII, the decrease is also more severe in the older group (−15.56 ± 8.29 vs. −47.13 ± 7.17%, *p* < 0.05). 

#### 2.2.2. Mitochondrial Calcium Retention Capacity

**Figure 3:**
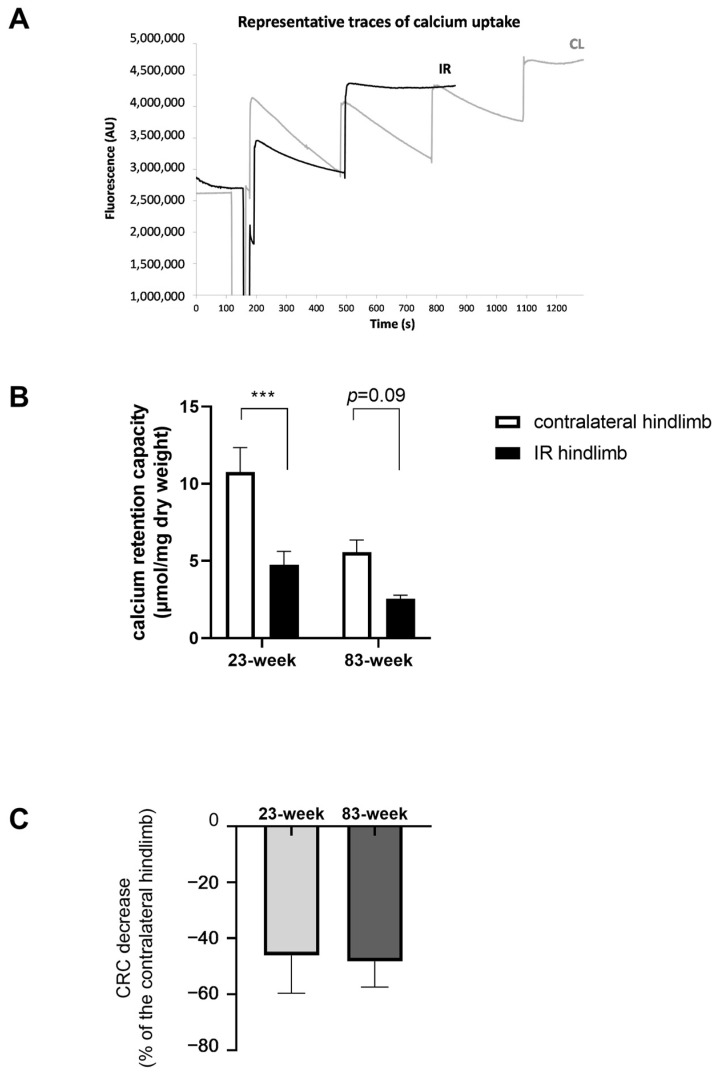
Effects of IR on calcium retention capacity in mDivi-1-treated young and old mice. (**A**) Representative traces of calcium uptake. (**B**) Calcium retention capacity (CRC) before mPTP opening. (**C**) Comparison of CRC between the 2 age groups (expressed in %). Results are presented as mean ± SEM. ***: *p* < 0.001. CRC: calcium retention capacity.

The resistance of mPTP opening in response to the calcium challenge was evaluated as shown in Figure 3. In the 23-week group, CRC is significantly altered in the ischemic hindlimb compared to the contralateral one, with a change of –55.8% (from 4.75 ± 0.87 to 10.76 ± 1.58 µmol/mg dry weight, *p* < 0.001). And, the CRC is altered in the 83-week group at the same level, −54.3%, but not significantly (5.57 ± 0.79 and 2.54 ± 0.24 µmol/mg dry weight for contralateral and ischemic hindlimb, respectively, Figure 3A–C).

### 2.3. Effects of IR on Oxidative Balance in mDivi-1-Treated Young and Old Mice

#### 2.3.1. Production of Superoxide Anion

**Figure 4:**
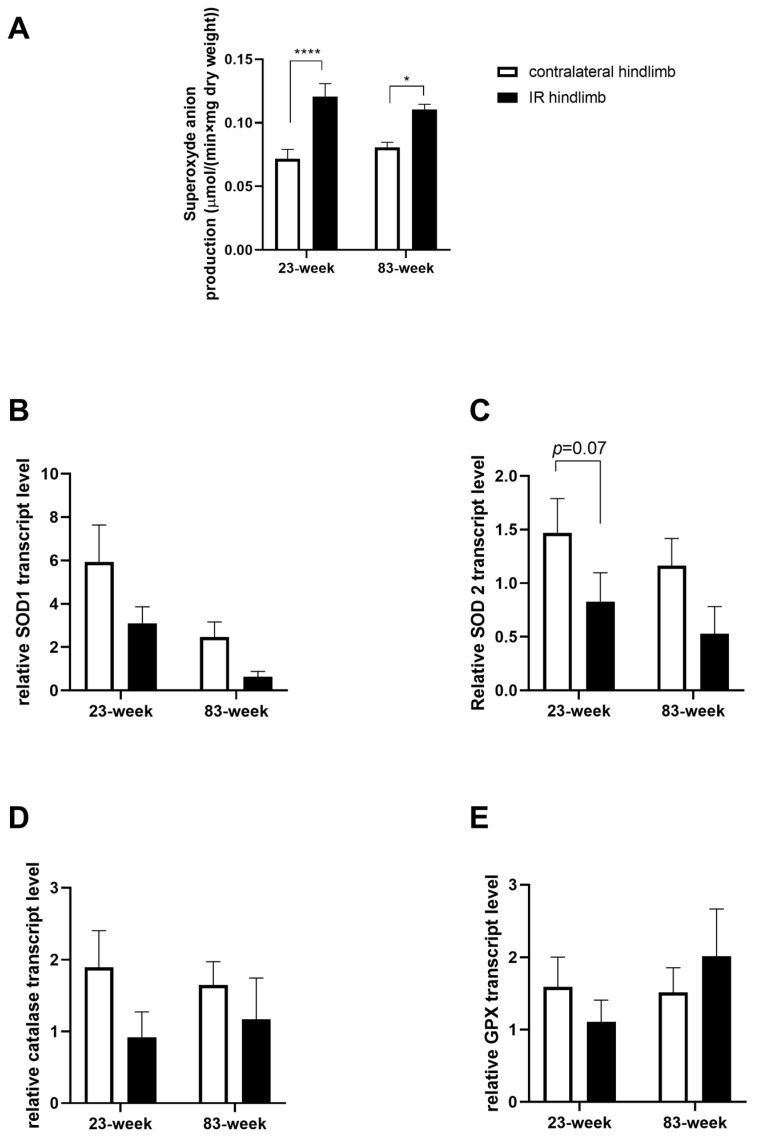
Effect of IR on superoxide anion production and antioxidant enzyme transcripts in young and old mice. (**A**) Superoxide anion production measured with electron paramagnetic resonance. (**B**) SOD 1 transcript level. (**C**) SOD2 transcript level. (**D**) Catalase transcript level. (**E**) GPx transcript level. Results are presented as mean ± SEM. *: *p* < 0.05; ****: *p* < 0.0001. SOD: superoxide dismutase. GPx: glutathione peroxidase.

In the 23-week group, IR increased the superoxide production in the IR hindlimb compared to the contralateral one: 0.12 ± 0.01 vs. 0.07 ± 0.01 µmol/(min × mg dry weight), *p* < 0.0001. A similar profile was observed in the 83-week group: 0.11 ± 0.01 vs. 0.08 ± 0.01 µmol/(min×mg dry weight), *p* < 0.05 (Figure 4A).

#### 2.3.2. Antioxidant System

Assessment of antioxidant enzyme transcripts showed no significant variation in superoxide dismutase (SOD) 1, in SOD2, catalase, and glutathione peroxidase (GPx) (Figure 4B–E). However, there was a downward trend for SOD2 (−44.2%, *p* = 0.07, and −54.3%, in the 23-week and 83-week group, respectively).

### 2.4. Effects of IR on Mitochondrial Dynamics in mDivi-1-Treated Young and Old Mice

**Figure 5:**
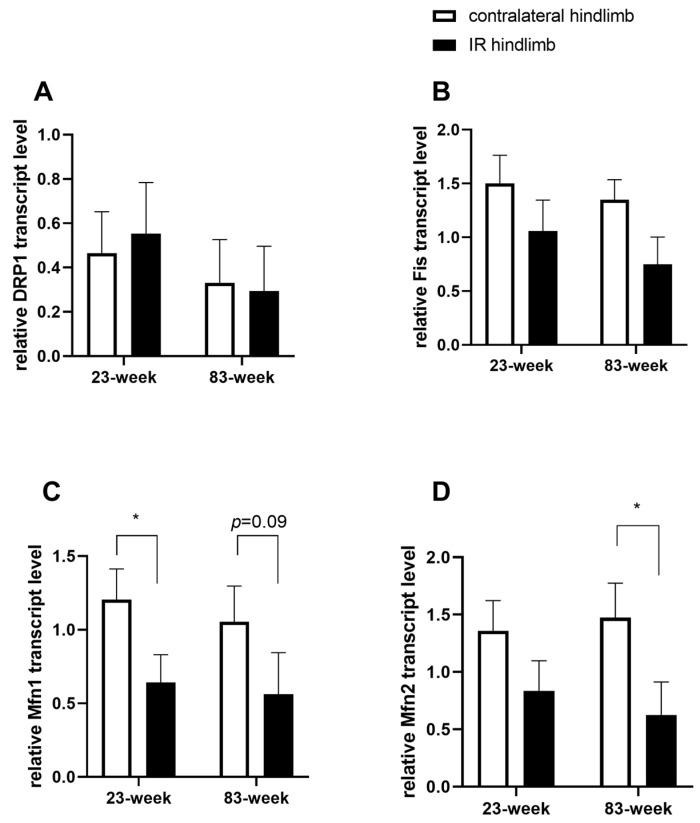
Effect of IR on mitochondrial dynamic in young and old mice. (**A**) Drp1 transcript level. (**B**) Fis1 transcript level. (**C**) Mfn 1 transcript level. (**D**) Mfn2 transcript level. Results are presented as mean ± SEM. *: *p* < 0.05. Drp1: dynamin-related protein 1. Fis1: mitochondrial fission protein 1. Mfn: mitofusin.

Study of RNA transcripts encoding for mitochondrial fission in *gastrocnemius* muscle was evaluated. Drp1 remained unchanged after IR (Figure 5A). On the other hand, another fission protein, Fis1 transcript tended to decrease in the two age groups (−30%, 1.50 ± 0.26 vs. 1.05 ± 0.28 for the 23-week group, and −44,4%, 1.35 ± 0.19 vs. 0.75 ± 0.25 for the 83-week group), but such decrease did not reach statistical significance (Figure 5B). 

RNA transcripts encoding for mitochondrial fusion, mitofusin (Mfn) 1, was decreased in the IR of the young group compared to the contralateral hindlimb (1.20 ± 0.21, 0.64 ± 0.19, *p* < 0.05). In the older group the decrease was not significant (1.05 ± 0.24, 0.56 ± 0.28). For Mfn 2, the RNA transcripts decreased significantly in the older group (1.47 ± 0.30, 0.62 ± 0.29, *p* < 0.05). At 23 weeks old, the RNA transcripts encoding Mfn 2 tended to decrease (1.36 ± 0.26, 0.83 ± 0.26).

## 3. Discussion

Besides confirming that aging enhances the deleterious effects of IR on muscle mitochondrial respiration, the main findings of this study are that IR-induced skeletal muscle mitochondrial dysfunctions (impaired mitochondrial respiration and reduced calcium retention capacity) were not corrected by mDivi-1 preconditioning, whatever the age. Further, mDivi-1 treatment did not oppose ROS production. At the transcript level, markers of antioxidant enzymes (SOD 1, SOD 2, catalase, and GPx) and fission markers (Drp1, Fis) remained unchanged or tended to be decreased in the ischemic leg. Fusion markers such as Mfn 1 or 2 decreased significantly. At the systemic levels, an IR-related increase in circulating lactate was still major despite mDivi-1 use.

### 3.1. Effect of Age on IR-Induced Deleterious Effects

Globally, the mitochondrial respiration characteristic in the non-ischemic muscle were similar to that previously reported in mice of similar age not treated with mDivi-1 [34]. This is consistent with the data reported recently by Kugler et al. showing that mDivi-1 did not modify the mitochondrial respiration in myotubes derived from obese human [36]. However, the decrease in mitochondrial respiration induced by IR was more severe in older mice, further supporting that aging favors greater lesion when muscle is submitted to IR [34,37]. 

This does not hold true when analyzing the CRC, but it might be because the CRC of the non-ischemic limb was lower in older mice, potentially blunting an IR-induced decrease. 

Concerning oxidative stress and mitochondrial dynamics, the values were similar in young and old mice, suggesting no or little effect of age per se on these parameters.

### 3.2. Inhibition of Mitochondrial Fission as a Therapeutic Option

In hindlimb IR, several therapeutic approaches targeting oxidative stress, inflammatory response, and calcium overload have been studied and demonstrated protective effects on skeletal muscle [19,38,39,40,41,42,43,44,45]. However, new therapeutic approaches still need to be open and growing evidence suggests that mitochondrial dynamics might be an interesting target to protect against IR injury since molecules inhibiting mitochondrial fission, including P110 and dynasore, improved mitochondrial functions [46,47,48,49,50]. In our study, we investigated a novel therapeutic approach through mitochondrial fission inhibition. Indeed, treatment with mDivi-1, a selective cell-permeable inhibitor of Drp1, inhibits the self-assembly of Drp1 by blocking Drp1’s GTPase activity and prevents apoptosis through inhibiting mitochondrial fission [51,52,53,54]. It showed protective effects against IR injury in other organs when it was administrated acutely, using variable dose and administration times. In vivo and in vitro studies demonstrated amelioration of mitochondrial functions and reduction in apoptosis and cell death after IR [25,28,29,55,56,57,58,59,60,61]. In several of them, mDivi-1 has been employed at a dose close to 50 mg/kg [24,26,27,49]. Accordingly, based on these previous data, we administered 50 mg/kg mDivi-1 1 h before ischemia induction.

### 3.3. No Protective Effect of mDivi-1 on Skeletal Muscle Ischemia Reperfusion-Induced Deleterious Effects

To the best of our knowledge, mDivi-1 treatment has never been investigated concerning the skeletal muscle mitochondrial function response to IR injury in young and aged mice. We here evaluated the effects of mDivi-1 on mitochondrial functions on the superficial *gastrocnemius* muscle (i.e., glycolytic muscle), known to be more sensitive to IR than oxidative muscles [62,63,64,65].

Interestingly, mDivi-1 did not protect against IR-induced mitochondrial respiration impairment. Indeed, as we previously reported in the same setting, an IR-induced decrease in mitochondrial respiration was similar without [34] and with mDivi-1. This was consistent with other studies, demonstrating that mDivi-1 had no protective effects on mitochondrial respiration, either after cytoplasmic irradiation [66], or after cerebral IR [67]. Also, Li et al. demonstrated that IR-induced renal dysfunctions were exaggerated with mDivi-1 treatment, particularly affecting mitochondrial complex I and apoptosis pathway [68]. 

Concerning mPTP opening, mDivi-1 reduced the intracellular calcium concentration increased by IR in the heart [59] Similarly, mDivi-1 can restore the cardioprotection of sevoflurane in a high glucose condition by inhibiting mPTP opening [69,70]. Ong et al. used pharmacological treatment with mDivi-1 to protect HL-1 cells from simulated IR and showed that inhibiting mitochondrial fission decreased mPTP opening susceptibility [25]. However, later, the same team reported a lack of cardiac protection against IR deleterious effects in large animals [31]. We also observed that mDivi-1 did not enhance the CRC after lower-limb IR. 

To go further, we investigated oxidative stress, which is considered as a key factor in IR-related muscular damage, and we determined both ROS production and the antioxidant defense. Superoxide anion is one of the main free radicals produced during IR, mainly by complex I and III of the mitochondrial respiratory chain [11,71]. Despite mDivi-1, IR increased ROS production significantly, as inferred from the increase in superoxide anion. Interestingly, and contrary to these results, mDivi-1 has also been shown to reduce ROS production and lipid oxidation and to increase antioxidant defense activity after IR in other organs, such as the heart and neurons [28,59,60], and in other in vivo and in vitro physio pathological conditions [49,72,73,74,75,76,77,78]. Possible mechanisms on the reduction in ROS production include increased activity of antioxidant defenses, such as SOD and/or improved mitochondrial respiration reducing ROS production at the source [79,80]. However, Rosdah et al. demonstrated on a simulated-IR injury model that the cytoprotective effect of mDivi-1 was not accompanied by changes in ROS production [58]. Kim et al. also demonstrated in differentiated 3T3-L1 adipocytes that mDivi-1 did not inhibit ROS production [81]. Similarly, we observed that the transcripts of the main antioxidant enzymes were not increased with mDivi-1 during IR. Thus, taken together, data are controversial and the relationship between mDivi-1 and ROS still deserves further study.

### 3.4. Effect of mDivi-1 on Mitochondrial Dynamics in the Setting of Lower-Limb Ischemia Reperfusion

Taken together, our study did not support a protective effect of mDivi-1 on skeletal muscle in the setting of IR. Noteworthy, mDivi-1 was not systematically beneficial, depending upon cell type, time of infusion, or duration of treatment (i.e., chronic administration) [66,67,68,82,83]. In fact, the mechanisms of mDivi-1 actions are not yet fully understood, and the specific inhibition of Drp1 activity by mDivi-1 is currently under debate.

mDivi-1 is generally described as a selective inhibitor of Drp1 on GTPase activity, inhibiting the self-assembly of Drp1 and, thus, mitochondrial fission [54]. To inhibit mitochondrial fission, Drp1 molecules assemble into a ring-like structure to constrict mitochondrial membranes in a GTP-dependent manner, and Fis1, anchored to the outer mitochondrial membrane, seems to participate in the recruitment of Drp1 through its cytosolic domain [84,85]. 

However, in our study, we demonstrated that mDivi-1 treatment had no effect on Drp1 and only tended to decrease mitochondrial fission protein, Fis1, transcript levels. This lack of significant Drp1 change does not preclude a potential action of mDivi-1 since mDivi-1 has been shown to attenuate skeletal muscle insulin resistance in obesity even if protein expressions of Drp1and Fis were not modified [36]. Thus, although acknowledging that a larger kinetic of transcript and protein levels of Drp1 might have been interesting, it is not certain that the acute characteristics of our study might allow change to be observed. There are many processes between transcription and translation, and the regulation and half-life of proteins is different from one protein to another, varying from minutes to days. As shown in the Ali and McStay’s review, the half-life of mitochondrial fission and fusion in humans are not reported [86,87].

Further, studies demonstrated that the actions of mDivi-1 inhibition on complex I activity, mitochondrial permeabilization, ROS production in neurons, heart, and fibroblasts can be fission-independent [69,72,80]. Bordt et al. suggested a reversible effect of mDivi-1 on complex I of the mitochondrial electron transport chain, potentially through ROS production modulation, but that was likely Drp1-independent [72]. Ruiz et al. demonstrated that mDivi-1 protects neurons against excite toxicity through Drp1-independent mechanisms, implying the modulation of mitochondrial function and intracellular calcium signaling [88]. Thus, further studies are required to clarify the pharmacokinetics, cytotoxic profiles, and the therapeutic potential of mDivi-1, especially on Drp1-dependent or independent mechanisms [83,89]. Of note, we observed that the mitochondrial dynamic balance was still impaired, since Mfn was decreased after IR, suggesting that modulating mitochondrial fusion might be interesting to perform. Indeed, although antagonists of Drp1 could likely reverse the atrophy observed during cancer-related cachexia [90], a concomitant deletion of Drp1 and Mfn 1 and 2 alleviated symptoms of cardiomyopathy and mice had better survival than when only Drp1- or Mfn 1-2 was deleted [91].

### 3.5. Limitations of the Study

Besides all the points debated before, another potential reason for the lack of skeletal muscle protection, like in another study [31], might be our study design. The use of multiple doses and of multiple time points for pharmacological conditioning with mDivi-1 might have been useful to totally rule out a potential protective effect of mDivi-1. Similarly, investigating the kinetic of eventual change in antioxidant activity, fission and fusion transcript and protein levels might have been interesting. However, the dose and timing chosen were based on the literature and in this specific setting of lower-limb IR, mDivi-1 failed to protect skeletal muscle mitochondrial functions.

Additionally, being the object of intense research, the precise mechanisms of mDivi-1 actions might not mainly rely on only mitochondrial fission in ischemic muscles, but this requires further studies.

## 4. Materials and Methods

### 4.1. Animals

Experiments were performed on male young and old C57Bl6J mice which were housed in a neutral temperature environment (22 ± 2 °C) on a 12 h light–dark cycle. Animals were fed with standard food and water ad libitum. All experiments were performed in agreement with the guidelines of the European Parliament on the protection of animals used for scientific purposes (Directive 2010/63/EU) and were approved by the ethics committee and the French Research Minister (agreement number 2018041811246867). 

### 4.2. Experimental Procedure and Muscle Sampling

Mice were anesthetized with a gas mixture of 4% isoflurane (Aerrane, CSP, Cournon, France) and oxygen in a ventilated hermetic cage, placed on heating blankets (Homeothermic blanket control unit, MINERVE, Harvard Apparatus^®^, Esternay, France) to maintain animal body temperature near 37 °C, and breathed spontaneously. 

**Figure 6:**
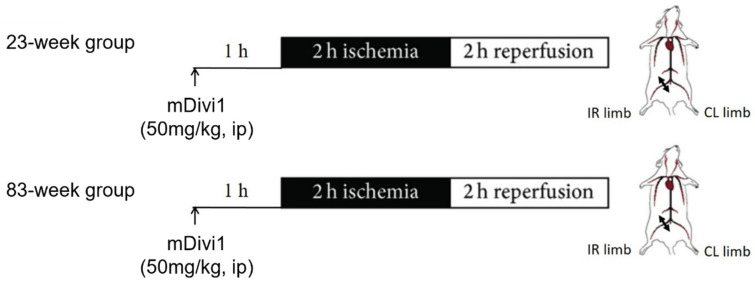
Experimental design.

A total of 14 mice were divided into two groups according to age (Figure 6). The first group was 23 weeks old (*n* = 7), and the second group was 83 weeks old (*n* = 7 initially but *n*= 6 for analysis since one mouse died at 1 h 20 min of reperfusion in this group). All mice were submitted to 2 h of ischemia with a tourniquet placed around the right hindlimb (IR), at the level of the groin, and 2 h of reperfusion. The contralateral (CL) hindlimb served as a non-ischemic control. mDivi-1 (50 mg/kg, Sigma M0199) was injected intraperitoneally 1 h before ischemia induction, as described previously [24,26,27]. Before ischemia, at the end of ischemia, and at the end of reperfusion, systemic lactates were measured in total blood obtained from the tail (lactate Pro device, LT710, Arkray^®^, KGK, Kyoto, Japan).

Top (23-week group): 23-week-old mice underwent 2 h unilateral hindlimb tourniquet ischemia (black bar), followed by a 2 h reperfusion (white bar). The left, non-ischemic contralateral (CL) hindlimb served as a control, and 50 mg/kg mDivi-1 was administered intraperitoneally (ip) 1 h before ischemia. Bottom (83-week group): the same protocol was performed on mice who were 83 weeks old. At the end of the reperfusion, *gastrocnemius* muscles (i.e., glycolytic), characterized by a greater susceptibility to IR-induced damage [63,92], from CL and IR limbs were harvested and immediately either placed in a Krebs-HEPES buffer (NaCl 99 mM, KCl 4.69 mM, CaCl_2_ 2.5 mM, MgSO_4_ 1.2 mM, NaHCO_3_ 25 mM, KH_2_PO_4_ 1.04 mM, D(+) glucose 5.6 mM, Na-HEPES 20 mM, pH 7.4 at 4 °C) for mitochondrial function evaluation, or frozen at −80 °C in liquid nitrogen to perform ribonucleic acid (RNA) analysis. 

### 4.3. Permeabilization of Skeletal Muscle Fibers

Samples were gently dissected on ice under a dissecting microscope for mitochondrial respiration and CRC measurements, and muscle fibers were permeabilized by incubation at 4 °C under stirring for 30 min in a buffer S (CaK_2_EGTA 2.77 mM, K_2_EGTA 7.23 mM, Na_2_ATP 6.04 mM, MgCl_2_ 6.56 mM, taurine 20 mM, Na_2_Phosphocreatine 12.3 mM, imidazole 20 mM, dithiothreitol 0.5 mM, K-methane sulfonate 50 mM, pH 7.0 at 4 °C) with saponin (50 µg/mL). Then, fibers were rinsed with agitation for 10 min at 4 °C in the buffer S. Using permeabilized fibers allowed preservation of the functional cellular environment and mitochondrial morphology [93].

### 4.4. Study of Mitochondrial Respiration by Oxymetry

Evaluation of oxygen consumption was performed using a Clark electrode in a thermostated oxygraphic chamber at 37 °C with continuous stirring (Oxygraph-2k, Oroboros instruments, Innsbruck, Austria). Briefly, 3–4 mg of wet weight fibers were incubated twice at 4 °C for 5 min with agitation in a buffer R+ (CaK_2_EGTA 2.77 mM, K_2_EGTA 7.23 mM, MgCl_2_ 1.38 mM, imidazole 20 mM, taurine 20 mM, dithiothreitol 0.5 mM, K-methane sulfonate 90 mM, Na-methane sulfonate 10 mM, glutamate 5 mM, malate 2 mM, K_2_HPO_4_ 3 mM, and bovine serum albumin 2 mg/mL, pH 7.0 at 22.1 °C). The permeabilized fibers were placed in 2 mL of buffer R+ in the oxygraphic chamber (V_0_). Then, a multiple substrate–inhibitor titration protocol was used before the addition of the saturating amount of adenosine diphosphate (ADP) (2 mM) (OXPHOS CI, also V_ADP_). Then, succinate was injected (25 mM) (OXPHOS CI + II). Finally, an injection of rotenone (0.5µM) inhibited the complex I (OXPHOS CII). The degree of coupling between oxidation and phosphorylation was evaluated by calculating the VADP/V0 ratio. Results were expressed as pmol/(s×mg wet weight).

### 4.5. Calcium Retention Capacity Evaluation in Ghost Fibers

CRC of skeletal muscle mitochondria under energized conditions allowed measuring the mitochondrial permeability transition pore (mPTP) opening. Briefly, permeabilized fibers (5–6 mg wet weight) were incubated at 4 °C for 30 min under stirring in buffer R+ containing KCl (800 mM) to extract myosin, block the calcium uptake by the sarcoplasmic reticulum, and, thus, allow calcium uptake only by mitochondria. Then, fibers were washed 3 times for 10 min in CRC buffer (Tris-Base 20 mM, saccharose 150 mM, KCl 50 mM, KH_2_PO_4_ 2 mM, and succinate 5 mM, pH 7.4 at 23 °C) containing bovine serum albumin (2 mg/mL) and ethylene glycol-bis (β-aminoethyl ether)-N, N,N′,N′-tetraacetic acid (EGTA) (5 μM).

Permeabilized ghost fibers were incubated in a quartz tank with continuous stirring at 24 °C in 1 mL of CRC buffer containing a calcium green-5N fluorescent probe (5 µM; excitation 500 nm; emission 530 nm). The reaction was started by the addition of a calcium pulse (20 mM), followed by calcium pulses every 5 min until it was necessary. After each pulse, a peak of extramitochondrial calcium was recorded and a rapid uptake by the mitochondria was observed, resulting in a decrease in extramitochondrial calcium concentration to a near-basal level. When mitochondria reached the maximal calcium loading threshold, the opening of mPTP happens and mitochondrial calcium is released, resulting in an abrupt increase in extramitochondrial calcium concentration. The amount of calcium necessary to trigger the mPTP opening was calculated from a standard curve relating calcium concentrations to the fluorescence of calcium green-5N. At the end of the experiment, muscle fibers were gathered, dehydrated at 150 °C for 15 min, and weighed. Results were expressed as µmol/mg dry weight. 

### 4.6. Reactive Oxygen Species Production Measurement by Electron Paramagnetic Resonance Spectroscopy

One of the best techniques used to detect the “instantaneous” presence of free radical species in the samples was the electron paramagnetic resonance spectroscopy. This technique consisted of oxidation from superoxide anion (O_2_.) and other ROS of a spin probe 1-hydroxy-3-methoxycarbonyl-2, 2, 5, 5-tetramethyl-pyrrolidine (CMH; oxidized form CM., Noxygen^®^, Elzach, Germany). Muscles were cut into 1–2 mm^3^ slices and incubated at 37 °C for 30 min in Krebs–HEPES buffer containing deferoxamine (25 μM), diethyldithiocarbamate (5 μM), and CMH (200 µM) in a thermoregulated incubator under a gas mix (O_2_: 2.7%, N_2_: 97.8%) and controlled pressure (20 mmHg) (Gas Treatment Chamber BIO-V and Temperature & Gas Controller BIO-III, Noxygen^®^, Elzach, Germany). Then, the reaction was stopped on ice and all experiment measures of CM. concentration were performed at 15 °C in disposable capillary tubes from 40 µL of supernatant, using the e-scan spectrometer (Bruker Win-EPR^®^, Elzach, Germany). Detection of ROS was conducted under the following settings: center field 3461.144 g, microwave power 21.85 mW, modulation amplitude 2.40 g, sweep time 5.24 s (10 scans), sweep width 60 g, and the number of lag curve points 1. The signal amplitude was calculated, and the concentration of CM. was determined from the standard calibration curve of CM. At the end of the experiment, muscle fragments were gathered, dehydrated at 150 °C for 15 min, and weighed. Results were expressed in µmol/(min×mg dry weight).

### 4.7. RNA Transcripts Encoding for Antioxidant Defense and Mitochondrial Dynamic

Transcripts encoding antioxidant enzymes (cytosolic and mitochondrial superoxide dismutases (SOD1 and 2, respectively), catalase, and glutathione peroxidase (GPx)), and mitochondrial fission and fusion (dynamin-related protein 1 (Drp1), mitochondrial fission 1 protein (Fis1), mitofusin 1 (Mfn1), and mitofusin 2 (Mfn2)) were analyzed. Two micrograms of RNA, isolated with TRIzol Reagent (Invitrogen, Life Technologies, Rockville, MD, USA), were converted to cDNA with SuperScript II reverse transcriptase (Invitrogen, Life Technologies, Rockville, MD, USA) and hexamer primers according to the supplier’s protocol. Quantitative RT-PCR was performed using the Light Cycler^®^480 SYBR Green I Master kit (Roche Diagnostics, Meylan, France) according to the supplier’s protocol. 18S was used for normalization.

Sequences of the primer sets used are listed in Table 1.

### 4.8. Statistical Analysis

Values are represented by mean ± SEM. Statistical analysis was performed using Prism 8.4.3 (Graph Pad Software Inc., San Diego, CA, USA). To determine the effect on systemic lactate, a Friedman test was used for values being paired at the time but did not follow a normal curve. For the analysis of the mitochondrial respiration decrease, a non-parametric test, the Mann–Whitney test, was performed. For all other comparisons, a two-way ANOVA followed by the Sidak’s multiple comparisons post hoc test was used to evaluate effects of IR and treatment in mice, taking age into account. A *p*-value of less than 0.05 was considered significant. 

## 5. Conclusions

The present study provides evidence that pharmacologic preconditioning with mDivi-1 is not protective on mouse skeletal muscle mitochondrial functions in this setting of lower-limb IR. Particularly, mDivi-1 did not oppose IR-induced mitochondrial respiration, CRC, and antioxidant defense transcript level impairments.

Many challenges and uncertainties remain to be addressed on pharmacological properties and the mechanism of action (Drp1-dependent or independent) before this molecule, or similar mitochondrial fission inhibitors, might be applied clinically. 

## Figures and Tables

**Table 1:** Primers used for real-time PCR.

Target Gene	Forward Primer 5′ → 3′	Reverse Primer 5′ → 3′
SOD1	CCAGTGCAGGACCTCATTTT	TTGTTTCTCATGGACCACCA
SOD2	ACCCAAAGTCACGCTTGATAG	GGACAAACCTGAGCCCTAAG
Catalase	CACTGACGAGATGGCACACT	TGTGGAGAATCGAACGGCAA
Glutathione peroxidase	TGCAATCAGTTCGGACACCA	AAGGTAAAGAGCGGGTGAGC
Drp1	AGAAAACTGTCTGCCCGAGA	GCTGCCCTACCAGTTCACTC
Fis1	CCGGCTCAAGGAATATGAAA	ACAGCCAGTCCAATGAGTCC
Mfn1	CCTCCATGGGCATCATCGTT	TGCAGCTTCTCGGTTGCATA
Mfn2	CTCAGGAGCAGCGGGTTTAT	GAGAGGCGCCTGATCTCTTC
18S	CGCGGTTCTATTTTGTTGGT	TCGTCTTCGAAACTCCGACT

## Data Availability

Data contained within the article.
